# A rare case of an irreducible Pipkin II fracture–dislocation of the femoral head in a young patient following low-energy trauma

**DOI:** 10.1016/j.ijscr.2020.04.005

**Published:** 2020-05-07

**Authors:** Marie Fernandez, Thomas Williams, Frédéric Dubrana, Rémi Di Francia

**Affiliations:** Service de Traumatologie, CHRU Cavale Blanche, Brest, France

**Keywords:** THA, total hip arthroplasty, CT, computed tomography, Femoral head fracture, Irreducible hip fracture-dislocation, Total hip arthroplasty, Case report

## Abstract

**Introduction:**

Fractures–dislocations of the femoral head are rare, and irreducible dislocations are even rarer. The functional prognosis is poor.

**Presentation of case:**

We report the case of a 23-year-old male brought to the emergency room following low-energy trauma. X-rays and a computed tomography scan revealed a Pipkin II fracture–dislocation of the right femoral head. We attempted reduction with the patient under general anesthesia; this failed, so we proceeded to total hip arthroplasty (THA).

**Discussion:**

A femoral head fracture has a poor prognosis; the principal complications are osteonecrosis, osteoarthritis, and heterotopic ossifications. No surgical treatment algorithm for a femoral head fracture (for fragment excision, followed by internal fixation or THA) is available, given the rarity of the injury and the lack of sufficient cases.

**Conclusions:**

We describe the mechanism of low-energy injury, as well as the controversial THA treatment chosen by this young patient.

## Highlights

• A rare presentation of hip fracture-dislocation: irreducible.
• Very unusual consequence of a low energy trauma mechanism.
• Damage appeared unusually in a young patient without known bone abnormalities.
• Controversial first line total hip replacement treatment.

## 1 Introduction

Fractures–dislocations of the femoral head are rare, and irreducible dislocations are even rarer. The functional prognosis is poor [1]. The surgical time required to reduce the dislocation creates a risk of long-term complications such as aseptic necrosis of the femoral head and late hip osteoarthritis [2,3]. The lesions are thus serious and are commonly caused by high-energy traumas such as traffic accidents and (more rarely) sports injuries [4]. Several classifications of hip dislocation are available. The Thompson–Epstein Type V dislocation [5] includes fracture of the femoral head; such fractures were divided into four subtypes by Pipkin [6]. Truly irreducible dislocations (i.e., the femoral head cannot be re-inserted into the acetabulum) must be distinguished from an incomplete reduction caused by a fragment of the femoral head or soft tissue interposition [5]. There is no optimal consensus treatment for irreducible dislocations with partial fracture of the femoral head. In young patients, any suggestion of first-line total hip arthroplasty (THA) remains controversial [7]. After obtaining informed consent of the patient, we here report the rare case of an irreducible posterior Pipkin II fracture–dislocation of the femoral head caused by low-energy trauma in a 23-year-old male. He underwent first- intention THA. This case report adheres to the SCARE checklist [8].

## 2 Presentation of case

A young male aged 23 years, a cook by profession, lacking any relevant medical or surgical history, was admitted to our emergency department following a fall during a jump from 60 cm height to a beach; he reported that he heard an audible crunch when he landed. This was a low-energy incident. He reported total and immediate functional impotence of the right hip.

**Fig. 1:**
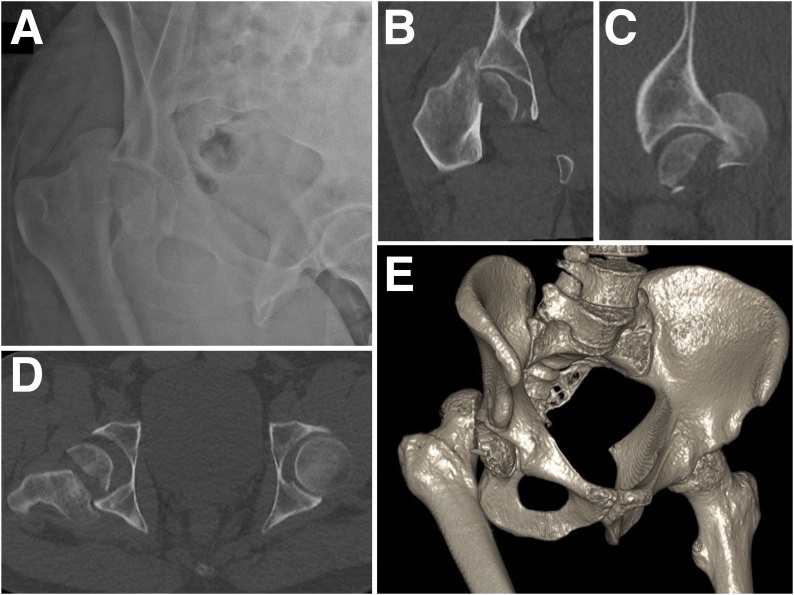
The initial anteroposterior (AP) pelvic X-ray (1A) and Pelvic CT images showing posterior dislocation and Pipkin II fracture of the femoral head, with a large fragment remaining in the acetabulum (1B: frontal view; 1C: sagittal view; 1D: axial view; 1E: three-dimensional reconstruction).

Before the trauma, his hip, femur, and knee were completely pain-free, his hip motion was full range and painless, and his Parker score was 9. He was hemodynamically stable, and respiration was normal. The pain was located opposite the right inguinal fold and was rated at 10/10 on a numerical scale. He was unable to move the right hip. Clinical examination revealed that the lower limbs were of different lengths. There was no neuronal deficit and a distal pulse was noted. There was no skin lesion. Pelvic X-rays revealed fracture of the right femoral head with posterior hip dislocation but without any associated acetabular fracture (Fig. 1A). Computed tomography (CT) confirmed posterior dislocation of the right hip and an enlarged type II Pipkin fracture of the femoral head. A cephalic fragment remained in the glenoid cavity, but a distal fragment had ascended and migrated posteriorly (Fig. 1B–E). The bone did not exhibit any intrinsic pathology.

**Fig. 2:**
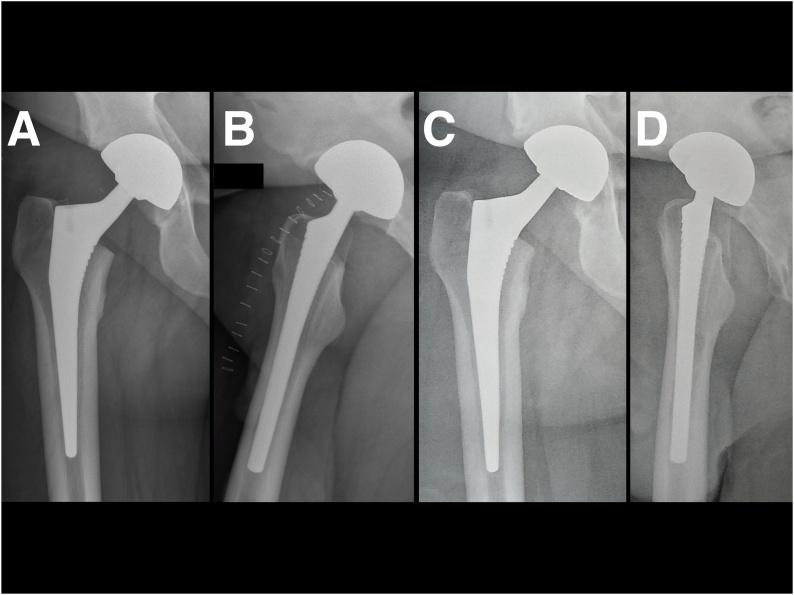
Immediate postoperative X-rays of the right hip after total hip arthroplasty (2A: AP view; 2B: profile view), and X-rays taken at the 12-month follow-up showing osseointegration without loosening (C: AP view; D: profile view).

The patient was admitted to the emergency department more than 2 h after the accident. The patient received paracetamol and morphine for analgesia. Because of the many patients in this department, X-rays and CT were available 5 h after the trauma, and general anesthesia could only be administered 9 h after the trauma due to occupancy of the emergency operating room. With the patient under general anesthesia with adequate curarization, we made several reduction attempts using external maneuvers, but these failed, probably because of the size of the bone fragment remaining in the acetabulum. We did not attempt open reduction using the Hueter or Smith–Peterson approach because of the high risk of devascularization and subsequent osteonecrosis of the femoral head. Confronted with a difficult situation, we decided to perform THA via a Moore posterolateral approach. This was particularly unfortunate because the location of the fractured portion (non-weight-bearing, inferior) suggested good clinical results after fixation. The operation was performed by a senior surgeon who was an experienced specialist in hip surgery at our center. Exploration triggered the (predictable) capsular burst secondary to dislocation and confirmed that the fracture involved 30% of the femoral head. We placed an uncemented dual mobility cup (Novae Sunfit© size 51 [Serf ©]) and an uncemented Corail© KHO size 11 stem (Depuy-Synthes©) with a ceramic head of diameter 28 mm + 5 (Fig. 2A, B). The immediate post-operative follow-up was simple. The control X-rays were satisfactory. The patient was allowed full weight-bearing postoperatively. He resumed walking the next day, using crutches. On discharge, the numerical pain scale was 0/10 and the Parker score 6.

The patient was pain-free at the 12-month follow-up. Right hip motion was identical to that of the contralateral hip, and the patient had resumed all activities of daily life. No infection or dislocation developed. The Parker and Harris (hip) scores were respectively 9 and 100. X-rays revealed good implant osseointegration without any signs of early loosening (Fig. 2C, D).

## 3 Discussion

A hip dislocation is a trauma emergency and reduction must be performed as soon as possible (within 6–8 h of trauma). A femoral head fracture–dislocation is rare, usually associated with posterior hip dislocation [[9], [10], [11], [12]]. A hip fracture–dislocation secondary to low-energy trauma (as in this case) is extremely rare [13]. A femoral head fracture has a poor prognosis [1,12]; the principal complications are osteonecrosis, osteoarthritis, and heterotopic ossifications [2,6,11]. Reduction attempts using external maneuvers (the Bohler technique) should be performed carefully because of the many possible complications, of which the most common is irreducibility [14] (maximum rate of 50% in the series of Duquennoy [15]).

If closed reduction is unsuccessful (fragments remain trapped in the joint), open reduction is necessary and opinions differ in terms of both the optimal approach and treatment of the cephalic fragment [[16], [17], [18]]. No surgical treatment algorithm for a femoral head fracture (dealing with fragment excision, followed by internal fixation or THA) is available, given the rarity of the injury and the lack of sufficient cases; no statistically significant data have been accumulated [10,19].

We decided to perform first-line THA given:

- the delay between trauma and management in the operating room (9 h) was longer than that recommended (6–8 h) to optimize the probability of a good outcome [7,9,10];
- the high complication rate (10–40%) following conservative treatment (osteonecrosis within 2 years and a 20% risk of osteoarthritis by 5 years [2,6,11,[16], [17], [18]]);
- the excellent revision rates (i.e., very low) of patients fitted with the THA dual-mobility cup [20,21], although these series do not include such young patients.

## 4 Conclusion

We report an irreducible posterior Pipkin II fracture–dislocation of the femoral head in a 23-year-old male. We thought it useful to describe the mechanism of low-energy injury, as well as the controversial THA treatment chosen by this young patient.

## Declaration of Competing Interest

All authors declare that they have no conflict of interest.

## Sources of funding

This research did not receive any specific grant from funding agencies in the public, commercial, or not-for-profit sectors.

## Ethical approval

Given the patient's consent to publish this case report, this study is exempt from the ethics committee of our institution.

## Consent

Patient consent obtained.

Statement included in the manuscript (p.6).

## Author contribution

M. Fernandez: data collection; literature review; writing.

T. Williams: operation, translation, review.

F. Dubrana: review.

R. Di Francia: design; review; translation; submission.

## Registration of research studies

researchregistry5297.

## Guarantor

Rémi Di Francia.

## Provenance and peer review

Not commissioned, externally peer-reviewed.
